# Prevalence and Incidence Estimation of HSV-2 by Two IgG ELISA Methods among South African Women at High Risk of HIV

**DOI:** 10.1371/journal.pone.0120207

**Published:** 2015-03-23

**Authors:** Irith De Baetselier, Joris Menten, Vicky Cuylaerts, Khatija Ahmed, Jennifer Deese, Lut Van Damme, Tania Crucitti

**Affiliations:** 1 Institute of Tropical Medicine, Antwerp, Belgium; 2 Setshaba Research Center, Soshanguve, Pretoria, South Africa; 3 FHI-360, Durham, North Carolina, United States of America; 4 Bill and Melinda Gates Foundation, Seattle, Washington, United States of America; UCL Institute of Child Health, University College London, UNITED KINGDOM

## Abstract

**Introduction:**

Previous comparison studies of the Kalon and HerpeSelect 2 ELISA IgG assays on sub-Saharan samples have found differences in the sensitivity and specificity of these assays. Using longitudinal samples from an HIV prevention study, we compared both assays and determined the HSV-2 prevalence and incidence in a South African young female population at elevated risk of acquiring HIV.

**Methods:**

Samples at baseline were tested in both assays using the manufacturers’ guidelines (cut-off > 1.10). When non-reactive in one assay, the final visit samples were tested to determine the incidence rate. Using correlation and regression analyses, the intra- and inter-assay variabilities were assessed.

**Results:**

The prevalence rate was 41.1% and 44.9% for Kalon and HerpeSelect using the manufacturer guidelines, respectively. Agreement between the two tests were high (kappa = 0.92). The original optical density values of both assays were highly correlated (R = 0.94), but the calibrator and correspondingly cut-off index values differed between the assays. Lowering the index value cut-off for the Kalon assay by 40% (to 0.66) resulted in a HSV-2 prevalence of 43.2%, and increased agreement between the assays (to kappa = 0.96). The incidence rate was 16.3/100 Person Years using the lower cut-off for the Kalon assay.

**Discussion:**

In this longitudinal study, we showed that the performance of the two assays was very similar. After lowering the cut-off for the Kalon assay to 0.66 early infections were detected without impairing its specificity. The prevalence and incidence rates are in line with previously described rates for sub-Saharan African cohorts.

## Introduction

Herpes simplex virus 2 (HSV-2) is one of the most prevalent sexually transmitted infections (STIs) globally and especially in sub-Saharan Africa.[1] Data on HSV-2 incidence in sub-Saharan Africa are not robust, but they suggest rates among women of 6 to 35 per 100 person years (PY).[2–7]

A number of epidemiological studies on HSV-2 have used the Kalon or HerpeSelect 2 ELISA IgG assays.[8] Both assays detect the type-specific glycoprotein gG-2, distinguishing HSV-2 from HSV-1. Studies in sub-Saharan Africa have shown that the HerpeSelect assay has better sensitivity but lower specificity than the Kalon assay and that the assay specificity varies by geographical location within the African continent and HIV infection status.[9,10, 11] The inferior specificity of the HerpeSelect assay may be due to cross-reactivity with other HSV types or with unidentified antibodies that might be common in African populations. Some researchers have suggested optimizing the cut-off index value (IV) of both assays to improve performance.[8,10–21] A meta-analysis of the performance of commercially available HSV-2 tests concluded that optimal performance in African populations may be obtained by increasing the cut-off for Kalon to 1.5 and that for HerpeSelect to 2.2–3.5.[8] It should be noted that most of the studies that suggest a revised cut-off for these assays comes from East-Africa and the need may be lesser for South-Africa.[13,17]

Antibody screening of HSV-2 is important, as many infected individuals who are not cognizant of their infection continue to shed HSV and may be an important reservoir for transmission.[22] The sensitivity and specificity of these assays can approach 100% when convalescent-phase serum, characterized by a high concentration of immunoglobulin G (IgG), is used.[23] However, the sensitivity of the assays may decrease when recent HSV-2 seroconversion samples are used.[15,24] This paper reports on a study nested in a randomized controlled trial of emtricitabine/tenofovir (FTC/TDF, Truvada) for pre-exposure prophylaxis for HIV infection among young African women (FEM-PrEP trial/ ClinicalTrials.gov #NCT00625404). [25] We determined the HSV-2 baseline prevalence and incidence among enrolled women in Pretoria, South Africa, and evaluated the performance of the Kalon and HerpeSelect HSV-2 serological assays using cross-sectional and longitudinal samples.

## Methods

### Participants and samples

The present study was performed retrospectively after the completion of the main study using stored serum specimens from women enrolled at the Pretoria site. Only samples from participants who contributed at least one follow-up visit and provided consent for future HIV-related research were included in this secondary analysis. The other sites were not included in this sub-study because the serum specimens were not stored at baseline or export approval could not be obtained.

Serum was collected in plastic uncoated serum separation tubes at screening (baseline) and at follow-up visits in weeks 4, 12, 24, 36, 52 and 56 and when clinically indicated. Samples were immediately taken to the on-site laboratory and processed within two hours of collection.[25] The on-site laboratory in Pretoria shipped the serum samples daily in temperature-monitored cool boxes containing ice packs to a private commercial laboratory in Durban for chemical testing and further storage at −20°C. After the end of the study, samples were shipped to the HIV/STI Reference Laboratory of the Institute of Tropical Medicine (ITM), Antwerp, Belgium.

### Laboratory methods

At the ITM, testing for HSV-2 antibodies was performed using two IgG ELISAs: the Kalon HSV-2 gG2 ELISA (Kalon Biologicals Ltd. Guildford, UK) and the HerpeSelect 2 IgG ELISA (Focus Technologies, Cypress, CA, USA). The assays were performed manually and according to the manufacturers’ instructions. All samples were tested in duplicate in both assays. The optical densities (OD) were read using a Bio-Tek ELx 800 microplate reader. For statistical purposes, all OD values above the quantification limit of 3 were assigned an OD value of 3. The geometric mean of the duplicate readings was used as the final OD, and the index values (IVs) were calculated as the final OD/calibrator OD. The obtained IVs were interpreted according to the manufacturers’ instructions; specifically, IV of <0.90 were classified as non-reactive, those of >1.10 as reactive and others as equivocal. If discordant results (reactive vs non-reactive) were obtained for duplicate specimens, the sample was retested and the geometric mean of the retested values was used for final interpretation. To assess incident HSV-2 infections, specimens collected at the final visit (i.e., after 52 weeks, or at the time of early discontinuation, or at the time of seroconversion among women newly infected with HIV) of women who were HSV-2 non-reactive at baseline in one or both assays were analyzed. If the participant was HSV-2 antibody reactive at her final visit on at least one assay, additional stored specimens were tested to estimate the time of HSV-2 seroconversion. Samples were tested retrospectively until non-reactive results were obtained with both assays.

### Statistical methods

Agreement between tests was assessed using Cohen’s weighted kappa statistic over the three possible result categories (non-reactive, equivocal or reactive). Intra-assay agreement of duplicate readings and inter-assay agreement were assessed using correlation and regression analyses of the log-transformed OD and IV.

Incident infections were defined separately for each assay. An "incident case" was defined as the transition of the test result from non-reactive (IV<0.90) to reactive (IV>1.10). The time of HSV-2 seroconversion was defined as the midpoint between the last non-reactive and the first reactive visit by assay. The median time to seroconversion was calculated using survival analysis methods (Kaplan-Meier). Those not reactive at the final visit were censored at that time.

### Ethics statement

The main clinical trial was approved by all applicable ethical and regulatory committees: The Protection of Human Subjects Committee (PHSC) of FHI360, the US Food and Drug Administration, the ethics committee (EC) of the University Hospital of Antwerp, the Institutional Review Board (IRB) of ITM, Medunsa Campus Research and EC (MREC) of South Africa, University of the Free State EC and Medicines Control Council of South Africa. In addition, separate approval was sought for this retrospective sub-study and received from the PHSC of FHI360, the EC of the University Hospital of Antwerp, the IRB of ITM and MREC of South Africa. Written informed consent was obtained from all participants prior to conducting any study procedures and only samples of participants who provided consent for future related HIV research were used for the present study. Study participant information was anonymized and de-identified prior to analysis.

Both studies were conducted in accordance with good clinical and laboratory practice guidelines.

## Results

### HSV-2 prevalence at baseline

Serum samples of 701 participants collected between August 2009 and November 2010 were tested at baseline. A total of 288 (41.1%) and 315 (44.9%) were identified as reactive results (IV>1.10) using the Kalon and HerpeSelect assays, respectively (Table 1). All samples reactive with the Kalon assay were also reactive with the HerpeSelect assay. Of the 27 specimens only reactive with the HerpeSelect assay, five showed equivocal results in the Kalon assay (Table 1).

**Table 1:** Baseline results for both assays using the manufacturer’s instructions (a) and the lowered cut-off of 0.66 for the Kalon assay (b).

**a**.		**HERPESELECT**			
KALON		Negative (IV<0.90)	Equivocal (0.90–1.10)	Positive (IV>1.10)	Total
	Negative (IV<0.90)	382	3	22	407 (58.1%)
	Equivocal (0.90–1.10)	0	1	5	6 (0.9%)
	Positive (IV >1.10)	0	0	288	288 (41.1%)
	Total	382 (54.5%)	4 (0.6%)	315 (44.9%)	
**b**.		**HERPESELECT**			
KALON		Negative (IV <0.90)	Equivocal (0.90–1.10)	Positive (IV >1.10)	Total
	Negative (IV <0.54)	382	3	8	393 (56.1%)
	Equivocal (0.54–0.66)	0	0	5	5 (0.7%)
	Positive IV (>0.66)	0	1	302	303 (43.2%)
	Total	382 (54.5%)	4 (0.6%)	315 (44.9%)	

IV = Index Value

### Intra-assay variability of the Kalon and HerpeSelect assays

In total, 1411 samples were tested using both assays and in duplicate. Using scatter-plots and Bland-Altman plots of the log_e_ OD values of the two repeats of each sample, we found that the HerpeSelect results (correlation coefficient R = 0.987) exhibited a very slight tendency to vary more between the two repeats of the same sample compared to the Kalon test (R = 0.994).

### Comparison of the Kalon and HerpeSelect assays

The inter-assay agreement (reactive, non-reactive, equivocal) at baseline was high (weighted kappa = 0.92).

**Fig 1:**
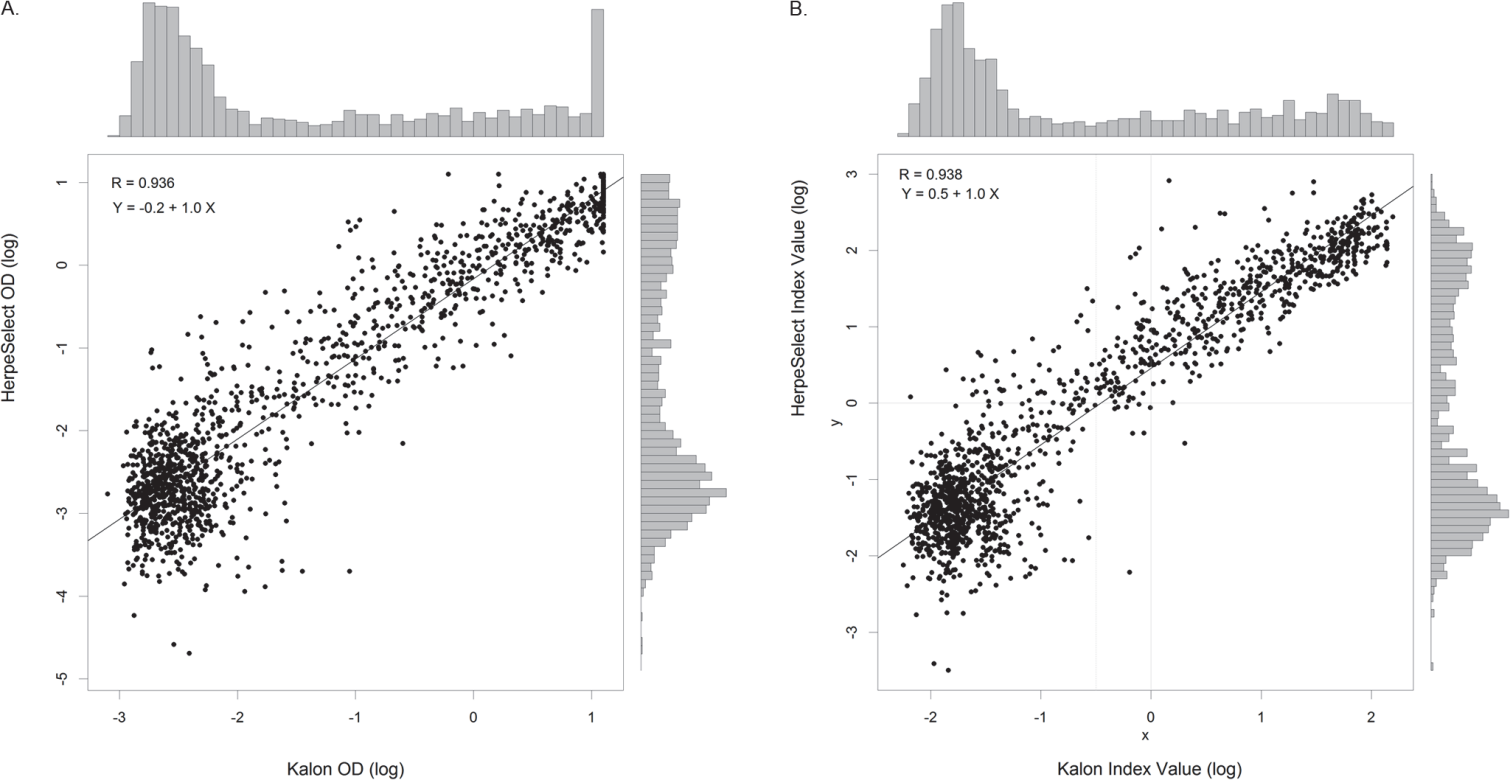
HerpeSelect vs Kalon assay results: a. optical densities (log), b. index values (log). Bold black line represents a linear regression line of Y (HerpeSelect result) in function of X (Kalon result). Full grey lines in panel b. represent theoretical cut-off of and index value of 1.00 for Kalon and HerpeSelect. The dotted line represents the lowered cut-off for the index value of Kalon at 0.60. Points in the upper right and lower left quadrant are concordant results, point in the upper left and lower right quadrant are discordant results at the respective cut-offs.

Comparing the continuous (log-transformed) OD and IV, the correlation between Kalon and HerpeSelect assays was high (R = 0.94). Of the 2822 individual OD values, 199 (8.0%) and 31 (1.2%) were above the quantification limit of the Kalon and HerpeSelect assays, respectively. Regression analysis indicated that the OD results for the Kalon assay were on average approximately 20% higher than the OD from the HerpeSelect assay (Fig. 1). However, the calibrator OD values were considerably higher for Kalon (median = 0.46, IQR = 0.39−30.52) than for HerpeSelect (median = 0.28, IQR = 0.23−0.29). Consequently, the IV, which is defined as the sample OD divided by the OD of the calibrator, tended to be 40% lower in the Kalon assay compared to the HerpeSelect assay. Lowering the IV cut-off for the Kalon assay by 40% to 0.60, corresponding to a 10% equivocal zone from 0.54 to 0.66 (compared to 0.90 and 1.10 following the manufacturer’s cut-off), resulted in more comparable HSV-2 prevalence estimates (43.2% and 44.9%, Table 1) and the agreement between the two assays increased (weighted kappa = 0.96).

### HSV-2 incidence

Follow-up specimens from all participants who were seronegative at baseline on either the Kalon or the HerpeSelect assay were tested using both Kalon and HerpeSelect. The estimated incidence rates were 14.0, 18.3 and 16.3 cases per 100 person years when using the Kalon assay with the manufacturer’s cut-off, the HerpeSelect assay with the manufacturer’s cut-off and Kalon assay with the revised cut-off of 0.66, respectively (Table 2).

**Table 2:** Estimated HSV-2 incidences for both assays using the manufacturer’s instructions and the lowered cut-off of 0.66 for the Kalon assay.

Test	Cut-off	Prevalence (%)	N at Risk	N with Positive FU	Incidence (%)	Incidence Rate (/100 PYR)
**Kalon**	Manufacturer ^†^	41.1	407	42	10.3	14.0
	Lowered Cut off ‡	43.2	393	47	12.0	16.3
**HerpeSelect**	Manufacturer ^†^	44.9	382	51	13.4	18.3

† Seroconversion = from a negative (<0.90) to a positive (>1.10) result.

‡ Seroconversion = from a negative (<0.54) to a positive (>0.66) result.

N at risk = susceptible (sero-negative at baseline); PYR = Person Year at Risk.

In total, 61 participants seroconverted, of which 45 were detected with both HerpeSelect and Kalon (IV>1.10), 9 with HerpeSelect and Kalon (IV>0.66) and seven with HerpeSelect only. Retrospectively, it was observed that of the 45 seroconverters, seven were actually HSV-2 seropositive at baseline using the lowered cut-off for Kalon (IV>0.66) and the HerpeSelect assay (IV>1.10). Furthermore, three were seroreactive using the HerpeSelect assay only (Table 3 and S1 Fig.).

**Table 3:** Results at baseline and final visit of the HSV-2 seroconverters using the manufacturers cut-off (IV>1.10) or the lowered cut-off (IV>0.66).

Baseline Result		Final Visit Result		
		H/K sero-positive (IV >1.10/>1.10)	H/K sero-positive (IV >1.10/>0.66)	H sero-positive/K sero-negative (IV>1.10/< = 0.66)
H/K sero-positive (IV >1.10/>1.10)	0			
H/K sero-positive (IV >1.10/>0.66)	7	7		
H sero-positive / K sero-negative (IV>1.10/< = 0.66)	3	3		
H/K sero-negative (IV< = 1.10/< = 0.66)	51	35	9	7

H = HerpeSelect assay; K = Kalon assay; IV = index value

We retroactively tested samples of all 61 incident cases to determine the time of seroconversion with both assays. In 25 subjects, the timing of seroconversion coincided in the two assays. Seroconversion was never detected earlier using the normal Kalon cut-off than the HerpeSelect assay. The lower Kalon cut-off identified one seroconversion one visit earlier (6 weeks) than the HerpeSelect (S1 Table). In the other cases, some delay occurred between seroconversion with the HerpeSelect assay and subsequent seroconversion with the Kalon assay. The median delay in seroconversion with the Kalon assay was seven weeks. When lowering the cut-off of the Kalon assay, the majority of seroconversions detected by the Kalon and HerpeSelect assays coincided, and the median delay was consequently 0 weeks.

### Longitudinal profiles of incident cases

**Fig 2:**
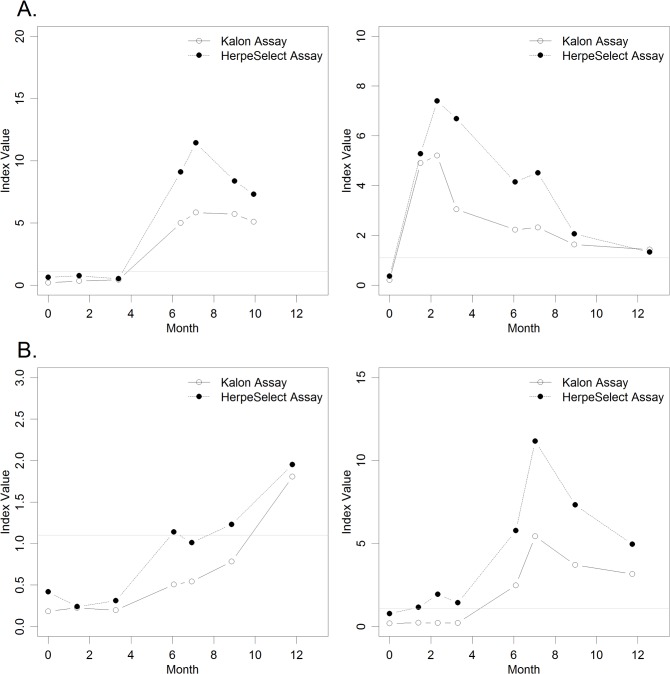
Longitudinal profiles. A. Incident profile with no delay in positivity for Kalon and HerpeSelect. B. Incident profiles with delayed increase of Kalon assay.

Select profiles are presented in Fig. 2 (additional profiles are available in S1 Fig. and S2 Fig.). Profile A is observed in the majority of the incident cases. The initial peak of positivity occurred at the same time for both assays. After the initial peak, the OD values decreased. Possible delayed positivity with the Kalon assay was only observed in the case of slowly rising OD, and only the IV appeared to be delayed (Profile B).

### HSV-2 co-infection among HIV seroconverters

Overall, 37 women acquired HIV at the Pretoria site during the study, three of whom were excluded from this analysis because they did not consent to future research. Of the 34 included, 17 were HSV-2 seropositive at baseline in both assays regardless of the cut-off used. In addition, one participant was reactive in the HerpeSelect and Kalon assays with lowered cut-off at baseline and became HSV-2 seropositive in the Kalon assay using the original cut-off at her HIV seroconversion visit (8 weeks after baseline). Of the 16 participants who were HSV-2 seronegative at baseline in both assays (IV>1.10), two became HSV-2 seropositive at the HIV seroconversion visit independent of the assay used and two became HSV-2 seropositive at the HIV seroconversion visit with the HerpeSelect assay, one of whom was also seropositive in the Kalon assay with the lowered cut-off.

Finally, of the 34 HIV seroconverters, 20 or 21 (58.8% or 61.8%) were co-infected with HSV-2 at the time of HIV seroconversion based on the manufacturer-suggested or lowered cut-offs, respectively. Using only the HerpeSelect assay, 22 HIV seroconverters were HSV-2 co-infected (64.7%).

## Discussion

In this study population of South African women at high risk for HIV, we found an HSV-2 prevalence of 41.1% and 43.2% and an incidence of 14.0 and 16.3 per 100 person years using Kalon cut-off IV of >1.10 and >0.66, respectively. This prevalence is in line with that of sub-Saharan African women (ranging from 30–80%).[26] A recent study on high-risk women in Mozambique found a HSV-2 prevalence of 60.6% at baseline and an incidence rate of 20.5% using only the HerpeSelect assay. The authors noted that their results might be overestimated.[4]

Although both HerpeSelect and Kalon EIAs are based on gG-2 using a similar expression, performance differences between the assays in terms of specificity and sensitivity were previously noted. In this longitudinal study, we found a high correlation and agreement between both assays, which was improved when using an adjusted IV cut-off for the Kalon assay. Both assays react in the same way but differ in operational procedure, the use of blanks and calibrator concentration. Looking at the intra-variability of both assays, we noticed that the HerpeSelect results tended to be slightly less robust than the Kalon results, which may result in part from the higher dilution factor used (1:101 vs 1:20), and the difference in blank (buffer vs air) The major divergence between the two assays is the composition and concentration of the assay calibrator. Based on the supplied calibrator, a cut-off IV of 0.60 with an equivocal zone of 0.54–0.66 for the Kalon assay should give results comparable to those of HerpeSelect. Previous studies suggested increasing the cut-off IV of the Kalon and HerpeSelect assays for sub-Saharan African samples to 1.50 and 2.20–3.50, respectively.[8] The difference in the cut-off between both assays in our analysis is 0.5 on a log scale (Fig. 1), which corresponds with the difference in the log values between the assays conducted in the above-mentioned study, which was 0.4 (cut-offs of 1.50 and 2.20) to 0.8 (cut-offs of 1.50 and 3.40). It should be noted that the setting of the cut-off is incorporated in the calibrator. Changing the cut-off at the level of the IV is somewhat circuitous, and it would make more sense to use an alternative calibrator. Previous studies used the Western Blot as gold standard and concluded that false reactive results of the HerpeSelect could be due to recent acquisition of HSV-2, the low sensitivity of the Western Blot, low levels of IgG and cross reaction with HSV-1. [8,13] A study conducted on African women with genital ulcer disease compared HerpeSelect and Kalon results with the results obtained by nucleic acid amplification on ulcer and cervicovaginal lavage samples and noted an excellent correlation (99%) between both assays for the negative and high positive samples but only moderate correlation (57%) for HerpeSelect IV between 1.10 and 3.50. Applying this higher cut-off IV, they would have misclassified 71% of the samples with an IV between 1.10 and 3.50.[31]

We also obtained reactive results with the HerpeSelect assay that may be false.[11,13] Of the 80 participants with a longitudinal profile, the samples of nine participants became reactive in the HerpeSelect assay and non-reactive in the Kalon assay over time, independently of the cut-off IV used. Most of the false reactive results obtained with the HerpeSelect assay had a very low IV (median = 1.77). We were not able to identify the cause of the false reactivity, but we cannot exclude the possibility that false negative results were obtained with the Kalon assay. False negative results may occur in patients with recent HSV infection,[28] patients with AIDS and recipients of solid-organ transplants.[29,30]

It has been demonstrated that the median time to seroconversion in patients with primary HSV-2 infection was 21 days for the HerpeSelect test and 120 days for the Kalon test.[24] Our findings are superficially in agreement, as we found that the Kalon assay became positive at a later time point for 36 out of the 61 incident cases. Using the revised cut-off for the Kalon assay, this number was brought down to 16 participants. Moreover, in one participant, the infection was detected at an earlier visit using the Kalon assay than the HerpeSelect assay. However, the delay in the detection of seroconversion with HSV-2 seems to be exclusively related to the difference in the calibrator constitution. Longitudinal assessment of the OD value showed that increases in the values obtained by the HerpeSelect and Kalon assays coincided.

The individual profiles of the majority of the incident cases showed an increase in IV (S1 Fig.), followed by a decrease, after which they remained stable. Of the 80 profiles, 16 participants show an interesting pattern of a sudden decrease in IV (S2 Fig.). The patterns in both assays are very similar for all participants. All of these participants would have been classified as HSV-2 seropositive using both assays at one time point, but 13 would have been considered HSV-2 negative in the Kalon assay at a later time point using the manufacturer’s cut-off, 6 of whom would have been considered not seropositive independently of the IV cut-off. The other three participants had Kalon equivocal results. In contrast, the HerpeSelect assay had IVs that remained reactive but decreased, and five became equivocal at a later time point. Seroreversion in the HerpeSelect assay has been previously documented in pregnant [32] and non-pregnant women[24,28,33] but could not be confirmed by Western blot analysis.[24,28] Seroreversion detection using the Kalon assay has not been described previously.

More than half (50.0%, 52.9% or 52.9% for Kalon IV>1.10; Kalon IV>0.66 or HerpeSelect IV>1.10, respectively) of the HIV seroconverters were HSV-2 co-infected at baseline and previous the HIV seroconversion visit. This finding highlights the need for multiple STI prevention tools, such as vaccines or microbicides. However, a recent study with tenofovir gel suggests that women with an active HSV-2 infection may be at significantly higher risk for acquiring HIV-1 due to enhanced ectocervical tissue susceptibility to HIV-1, and the effect of the tenofovir gel on HIV acquisition may be lower.[27] Therefore, caution should be warranted and additional studies are required.

To our knowledge, this is the first longitudinal study comparing the HerpeSelect and Kalon assays over time for South-Saharan African samples. The study benefited from a large sample size and extended follow-up; however, a number of limitations exist. The study was restricted to samples collected from participants at only one study site. Due to the lack of a reliable gold standard, the final classification of the discordant results at the final visit will remain unknown. We cannot exclude that false reactive or non-reactive results have been obtained in both assays. However, none of the participants seroreverted to HSV-2 negative in both assays. Another shortcoming is the fact that we did not censor the participants who missed visits and therefore had a longer period of time between blood samples. Due to budget constraints, we used a retrospective procedure and acquired longitudinal data restricted to the incident cases and to participants who had discordant results at baseline or at the final visit.

In conclusion, we demonstrated that both Kalon and HerpeSelect EIAs perform in the same way and generally provide concordant results. However, the use of an adjusted cut-off IV >0.66 or, preferably, a modified calibrator for the Kalon assay will give more concordant results relative to the HerpeSelect assay. False reactive results were found with the HerpeSelect assay; however, in contrast to many other studies [8,10–12,14–21], we did not consider increasing the cut-off IV of the HerpeSelect assay, as this would result in the misclassification of 14 participants at baseline who were in fact already HSV seropositive, as revealed by the follow-up data. After statistical analysis, we decided to lower the cut-off IV of the Kalon assay to 0.66 because intra-assay analyses showed that the Kalon assay is the most robust, no false reactive results were found and earlier infections were identified with the lowered cut-off

## Supporting Information

S1 Fig Individual profiles of 61 incident cases. (PDF)

S2 Fig Individual profiles and index value details of participants who had a sudden decrease in index values. (PDF)

S1 Table Time of seroconversion for HerpeSelect and Kalon assay using manufacturer’s cut-off and lowered cut-off. (PDF)
