# Supplementary material for: Prevalence and Incidence Estimation of HSV-2 by Two IgG ELISA Methods among South African Women at High Risk of HIV
Source: PLoS One. 2015 Mar 23;10(3):e0120207. doi: 10.1371/journal.pone.0120207 (PMC4370866; doi:10.1371/journal.pone.0120207)

S1: Individual profiles of 61 incident cases

A. Incident cases for HerpeSelect (IV>1.10) and Kalon assays (IV>1.10) using manufacturer's cut-off (n = 35)

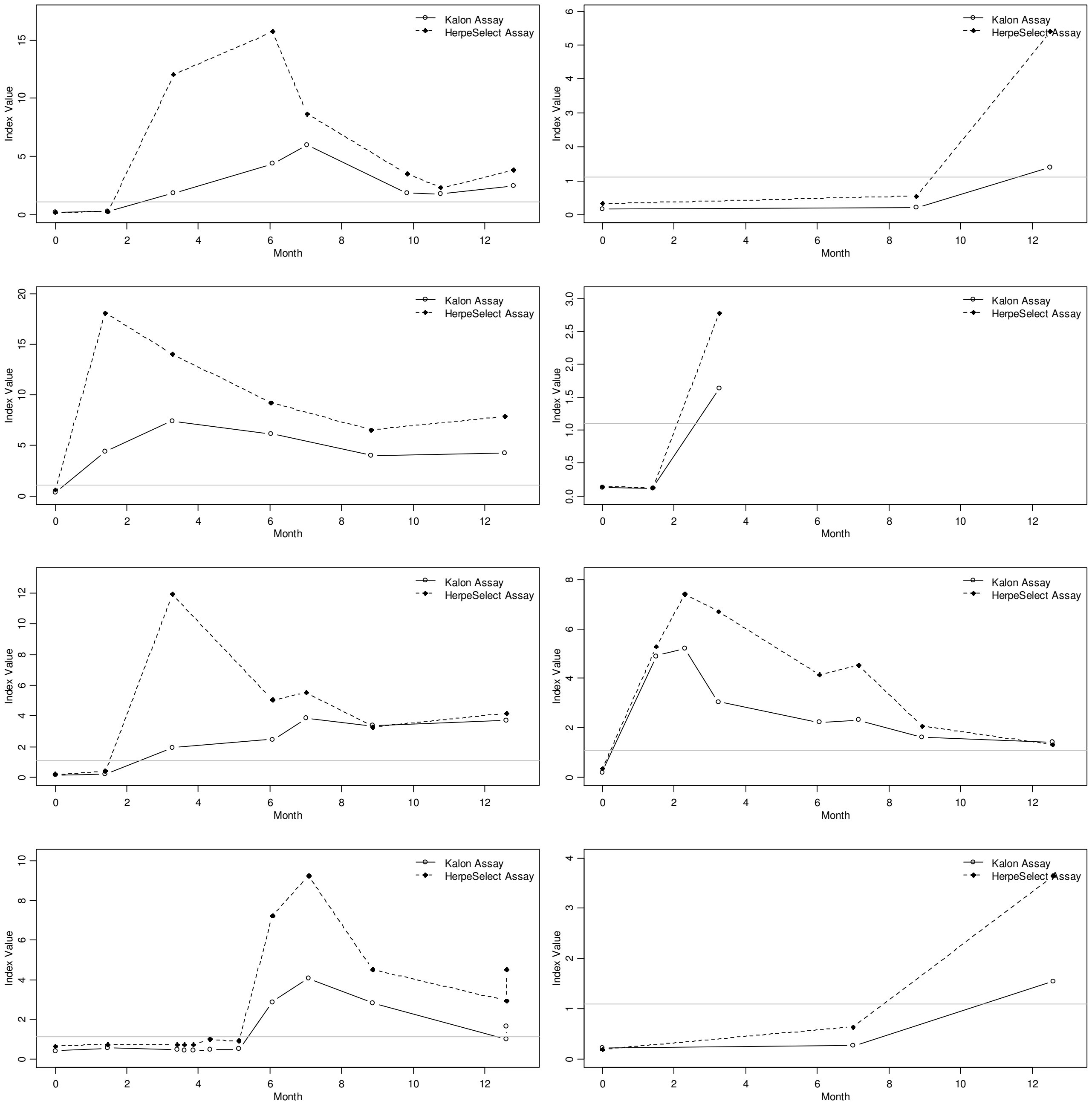

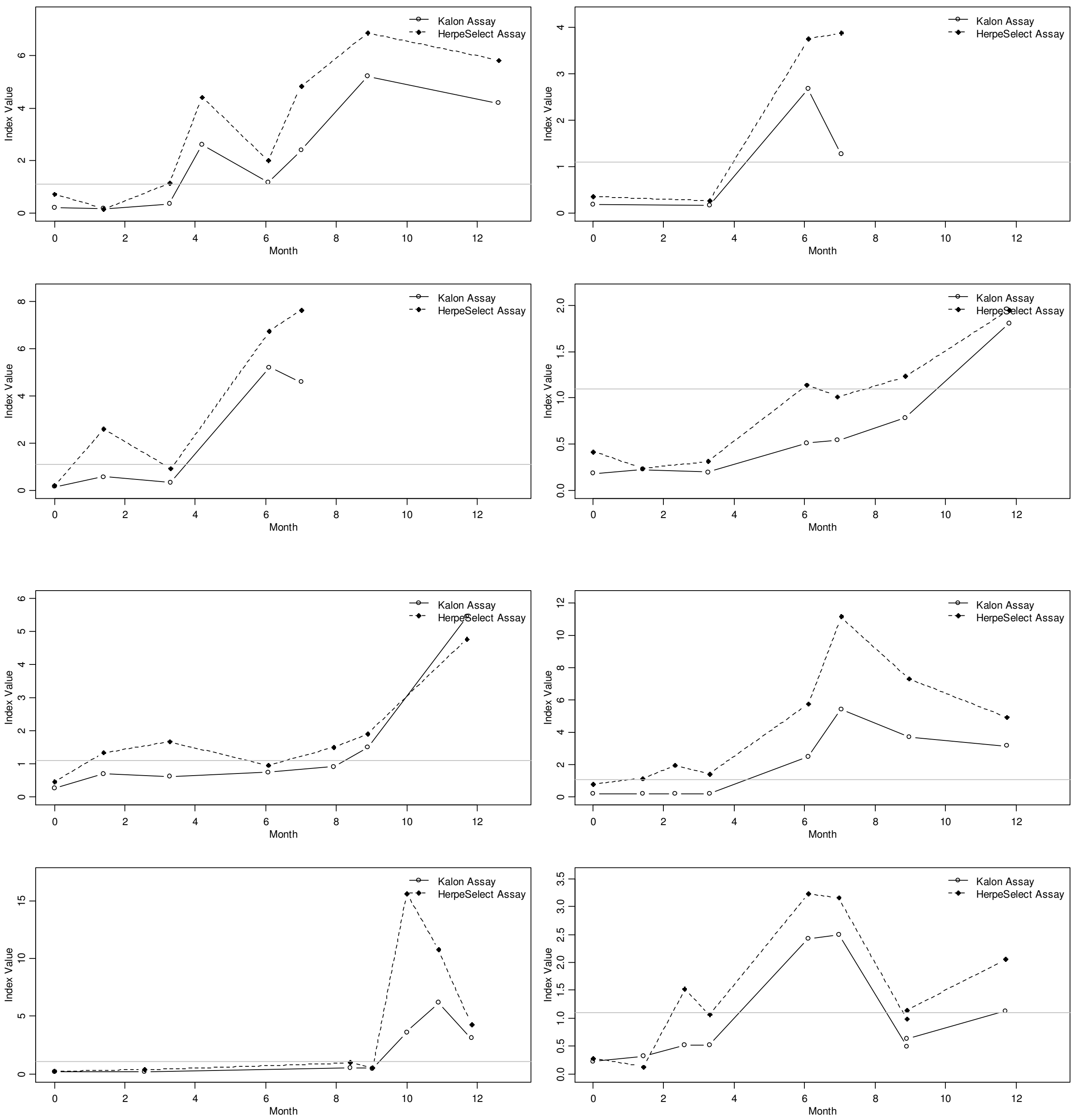
Kalon Assay
HerpeSelect Assay
Index Value
Month

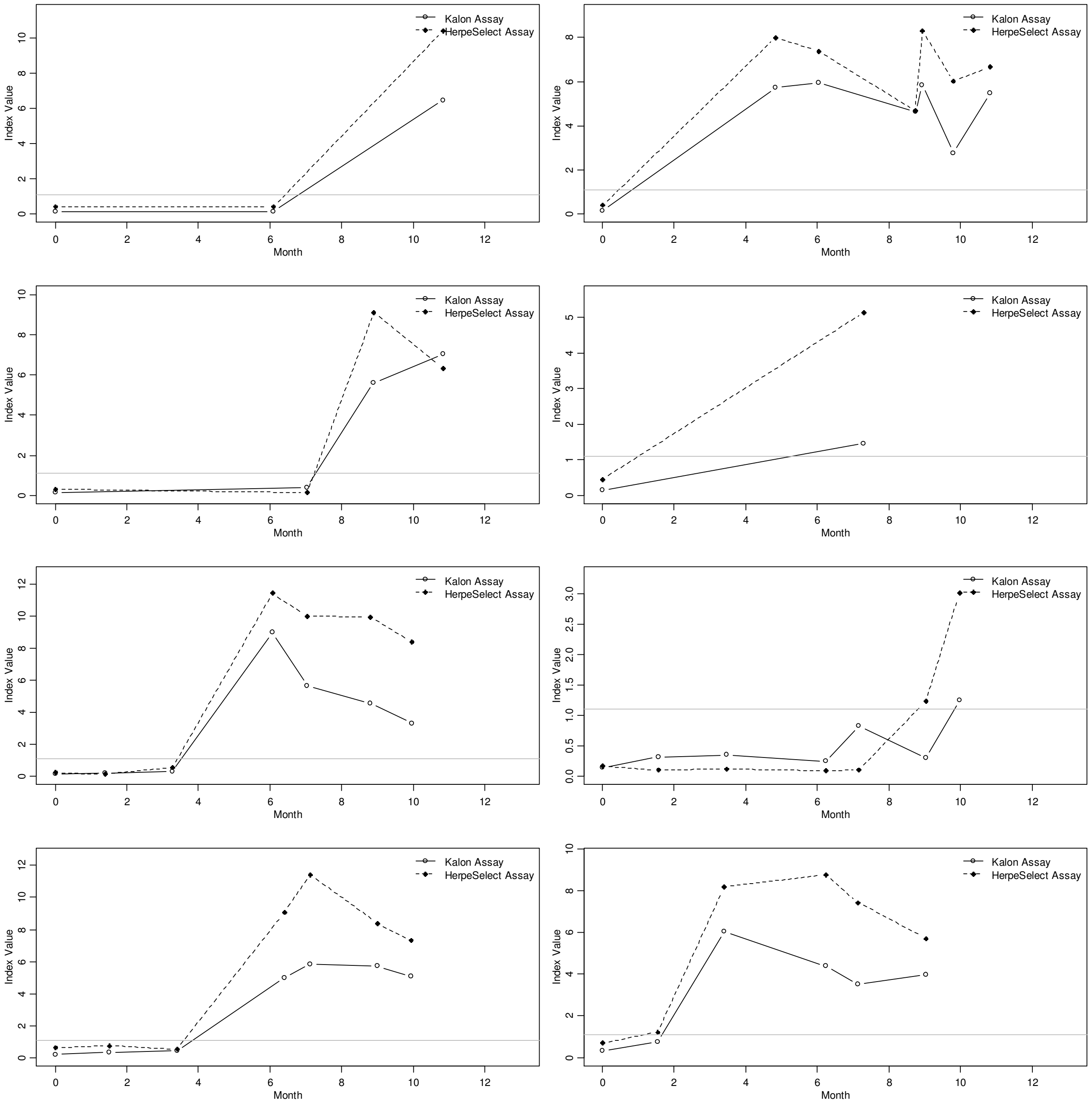
Kalon Assay
HerpeSelect Assay
Index Value
Month

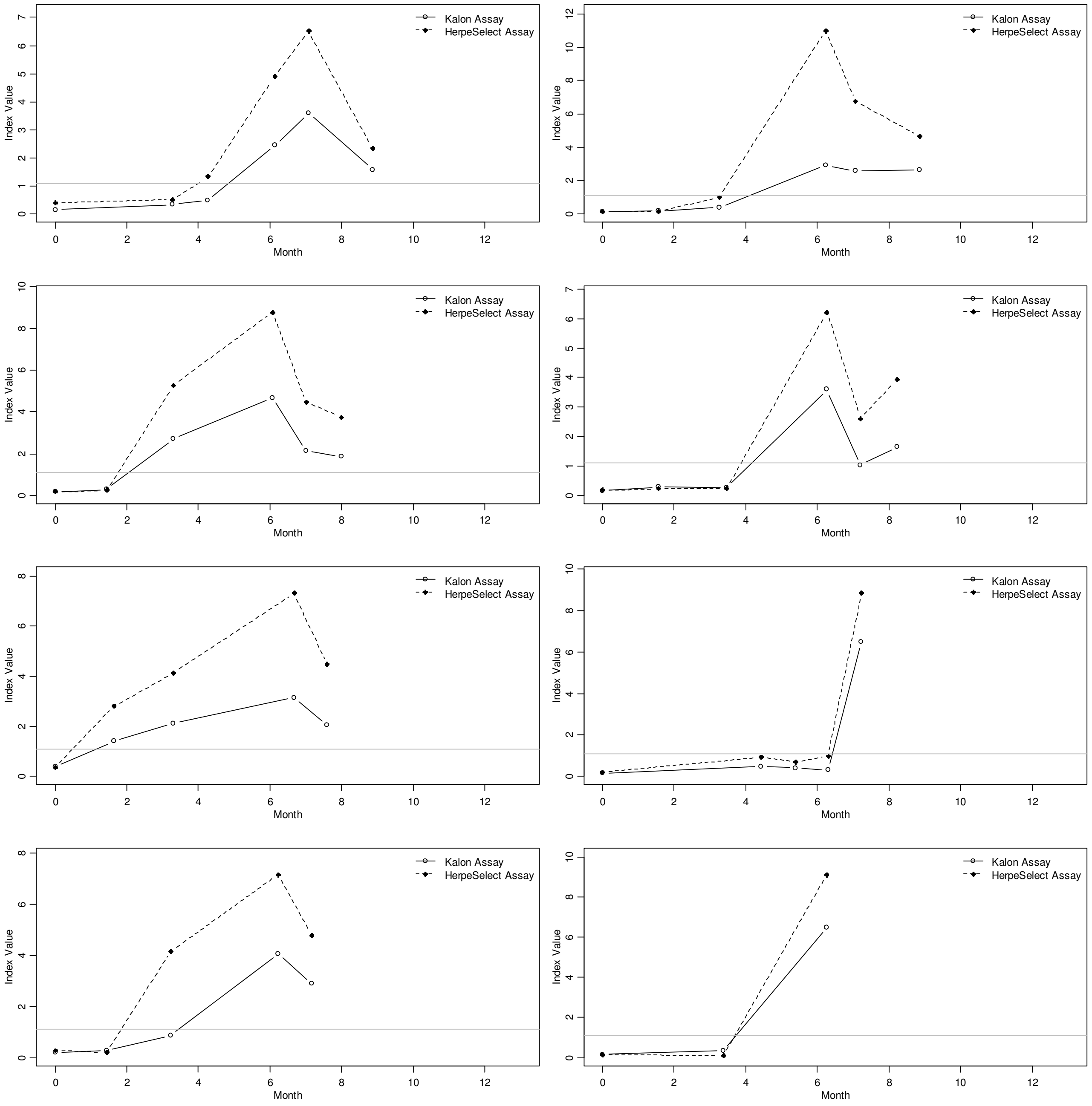

Kalon Assay
HerpeSelect Assay
Index Value
Month

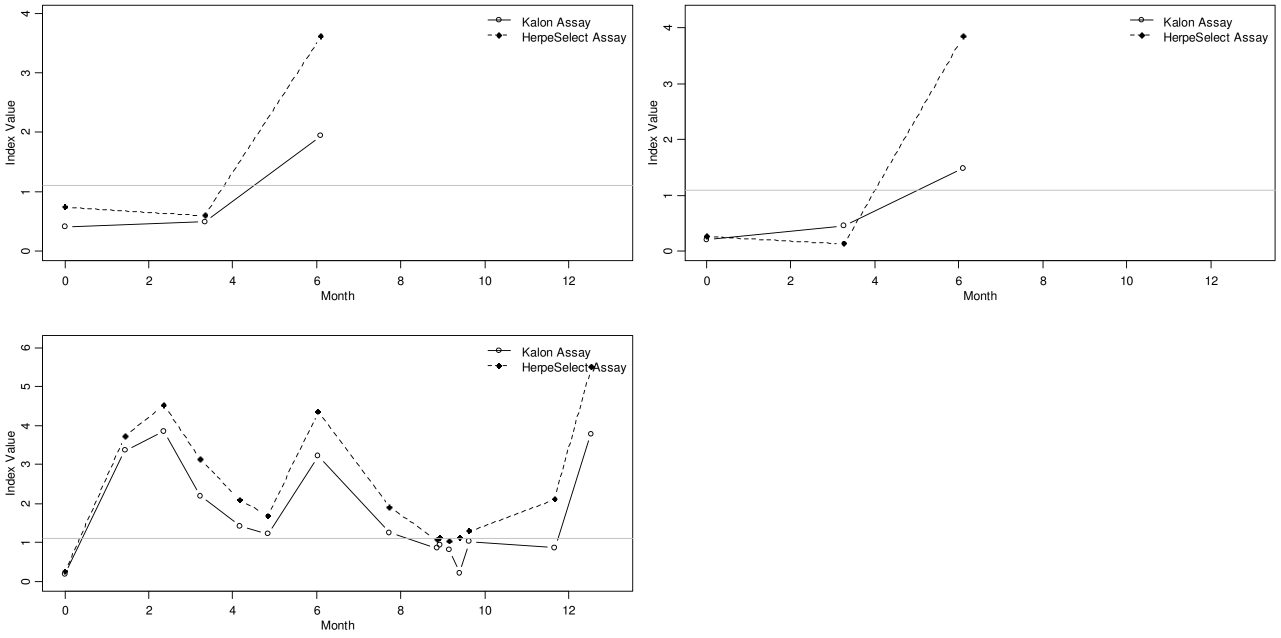
Kalon Assay
HerpeSelect Assay
Index Value
Month
0
2
4
6
8
10
12
0
1
2
3
4
5
6

B. Additional incident cases with HerpeSelect (IV>1.10) and Kalon assays using lowered cut-off (IV>0.66)
(n = 9)

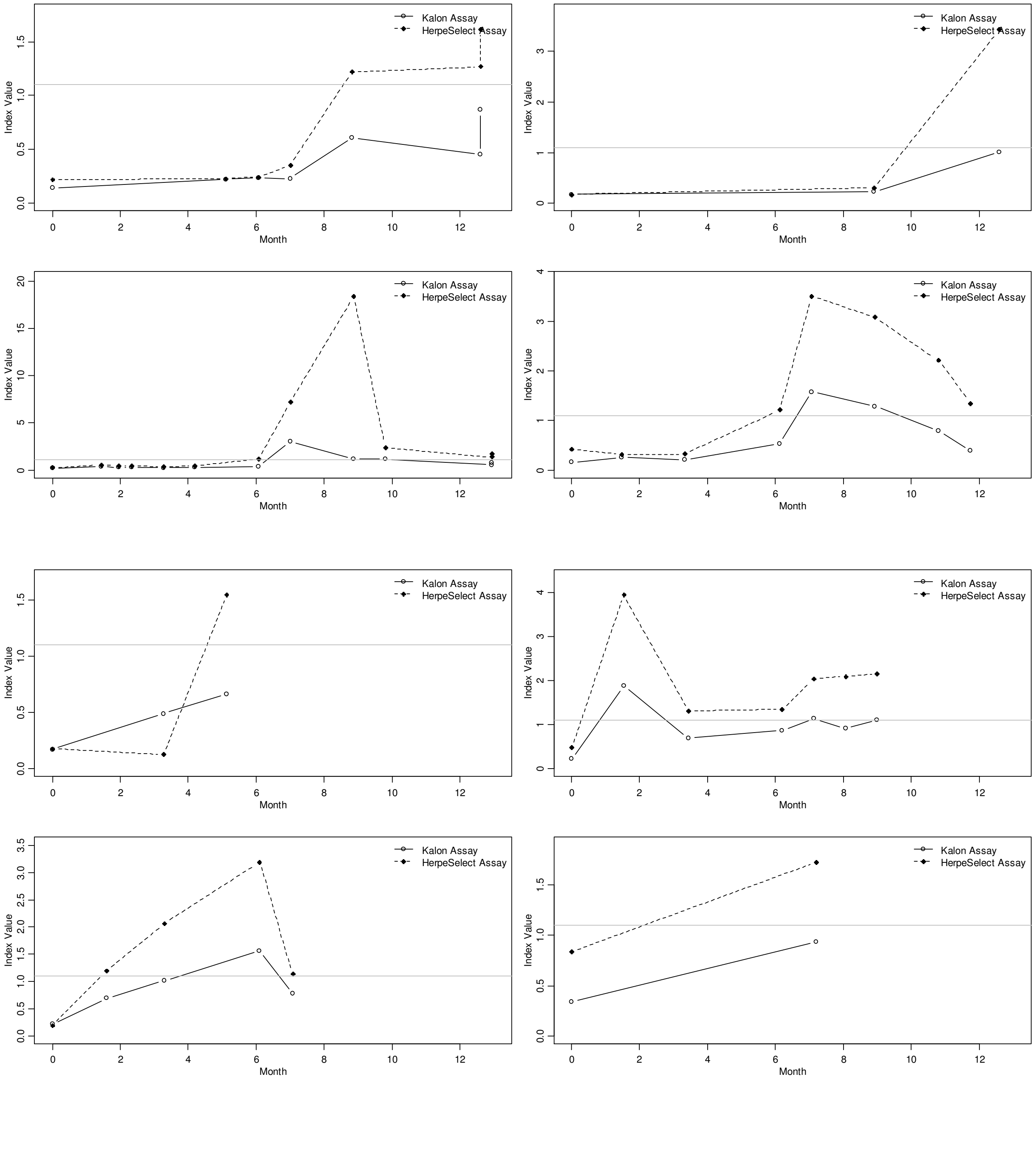

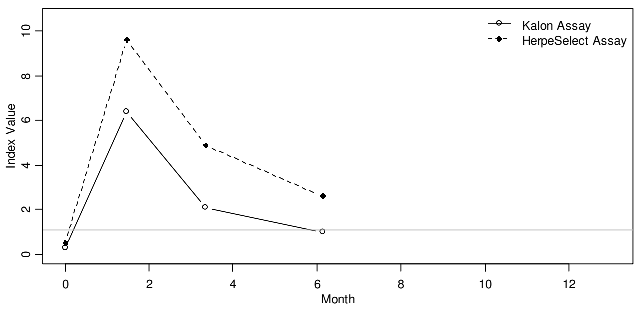

C. Incident cases only with HerpeSelect assay (IV>1.10) (n=7)

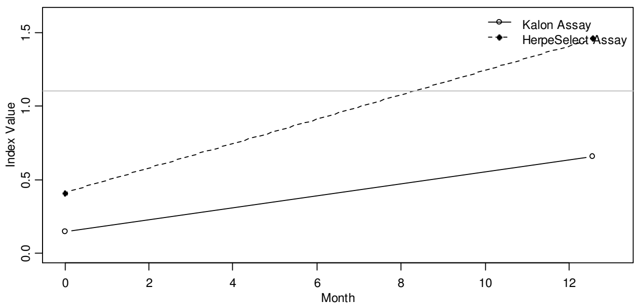

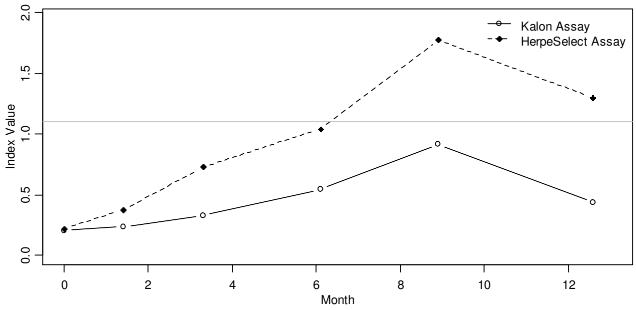

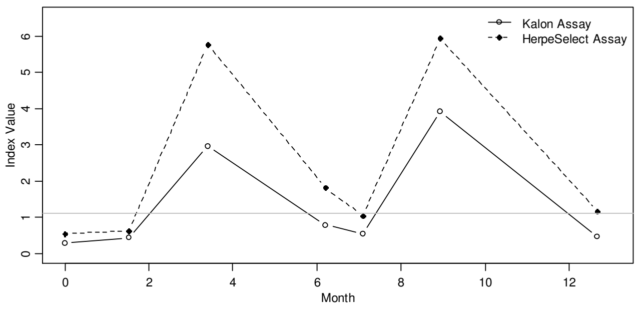

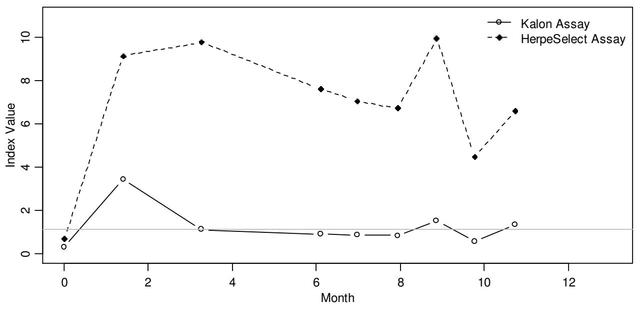

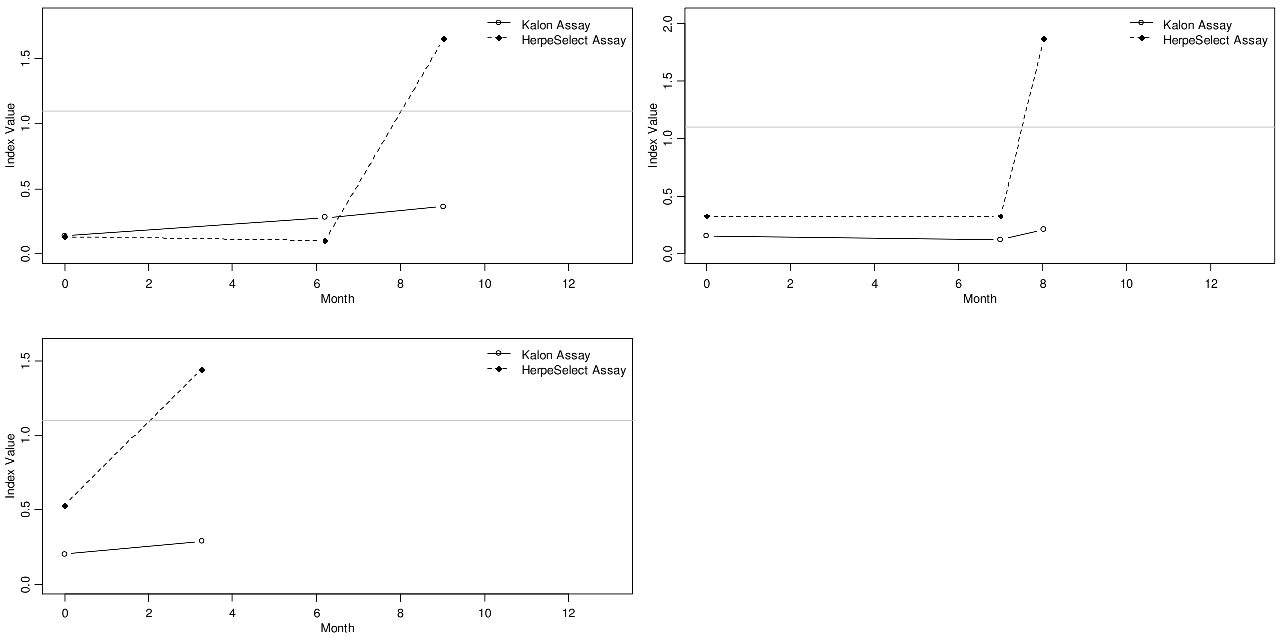

D. Cases that were found to be not HSV-2 sero-negative at baseline using HerpeSelect (IV>1.10) and Kalon using lowered cut off (IV>0.66) (n = 7)

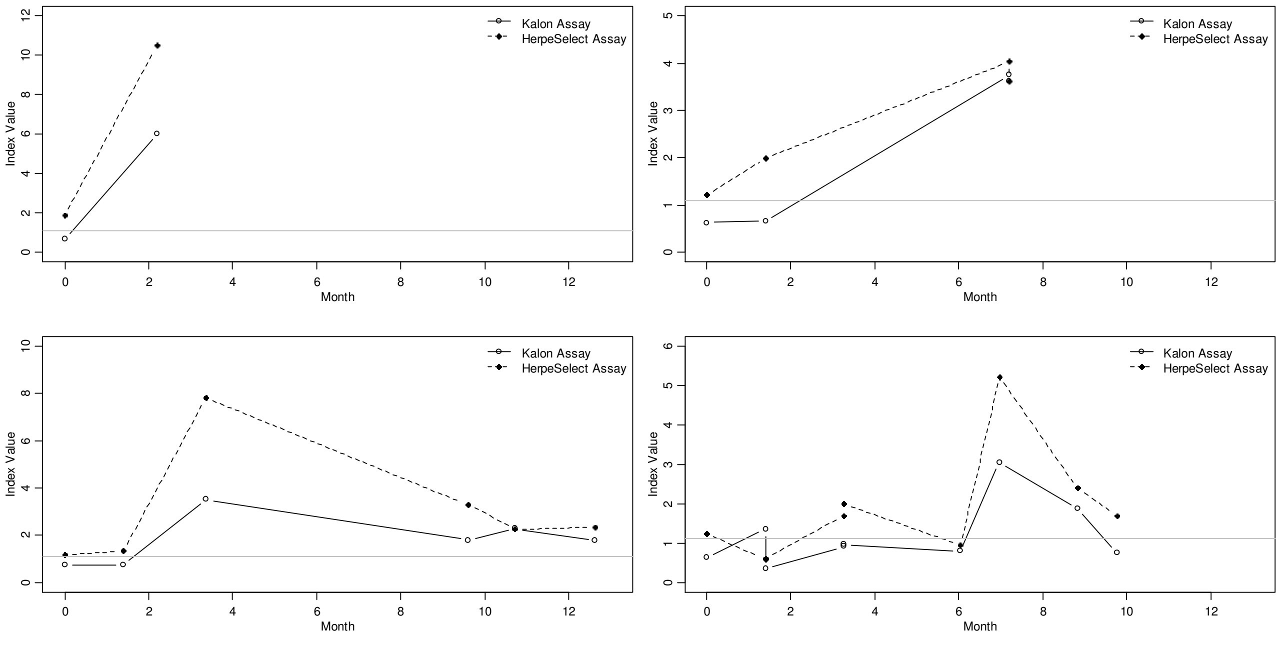

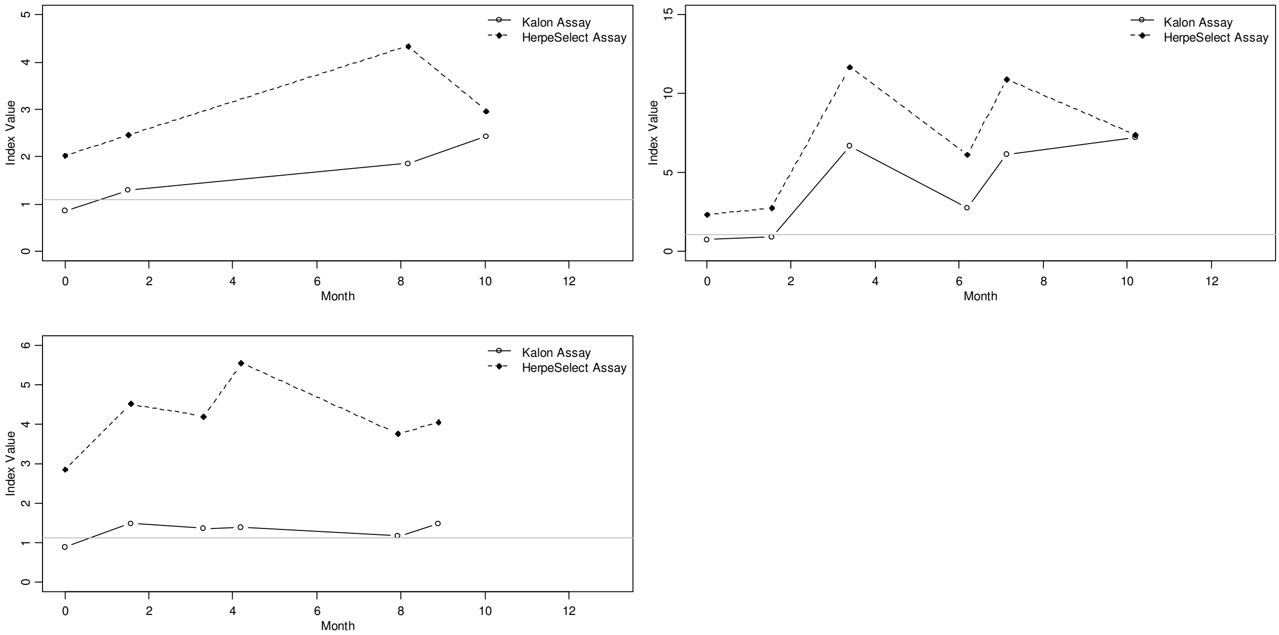

E. Cases that were found to be not HSV-2 sero-negative at baseline using only HerpeSelect (IV>1.10) (n=3)

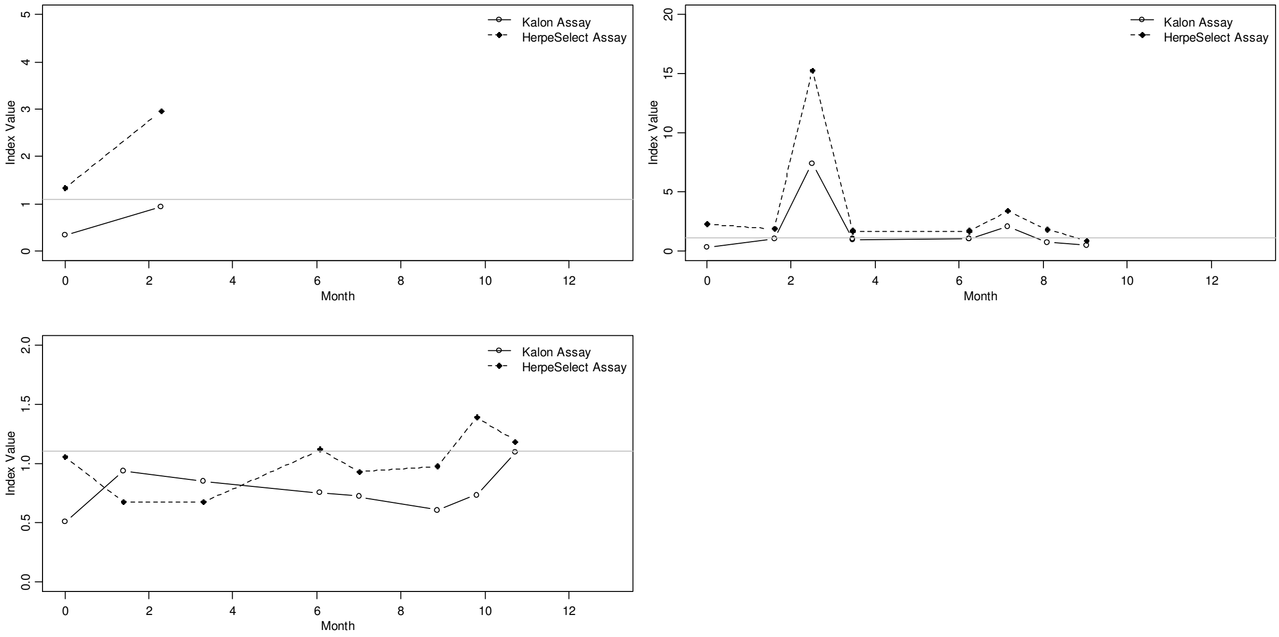

Supplement: S1 Fig — (PDF) [file pone.0120207.s001.pdf]
