# Supplementary material for: Prevalence and Incidence Estimation of HSV-2 by Two IgG ELISA Methods among South African Women at High Risk of HIV
Source: PLoS One. 2015 Mar 23;10(3):e0120207. doi: 10.1371/journal.pone.0120207 (PMC4370866; doi:10.1371/journal.pone.0120207)
Supplement: S2 Fig — (PDF) [file pone.0120207.s002.pdf]

S3: Individual profiles and index value details of participants who had a sudden decrease in index values (n = 16)

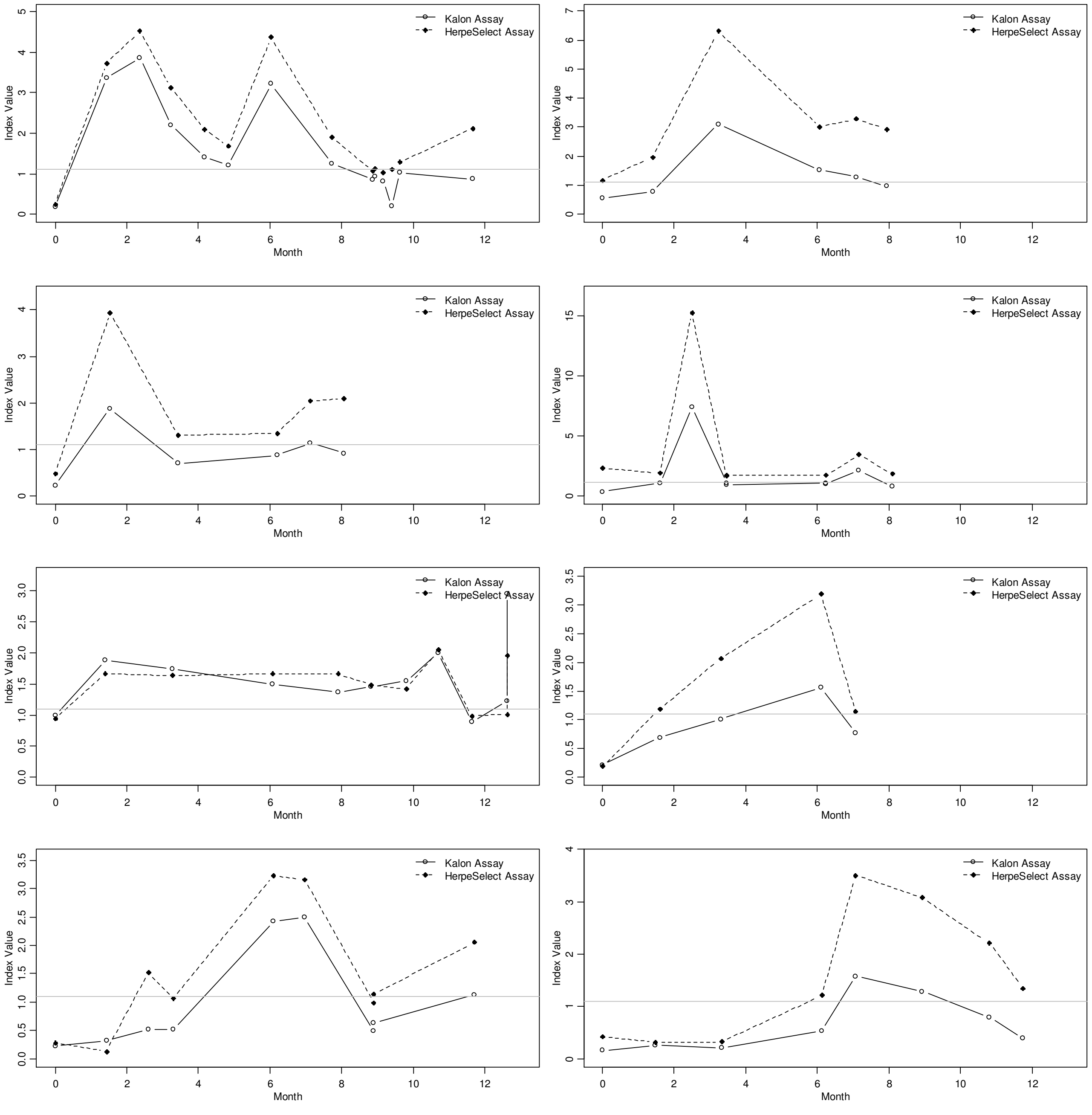

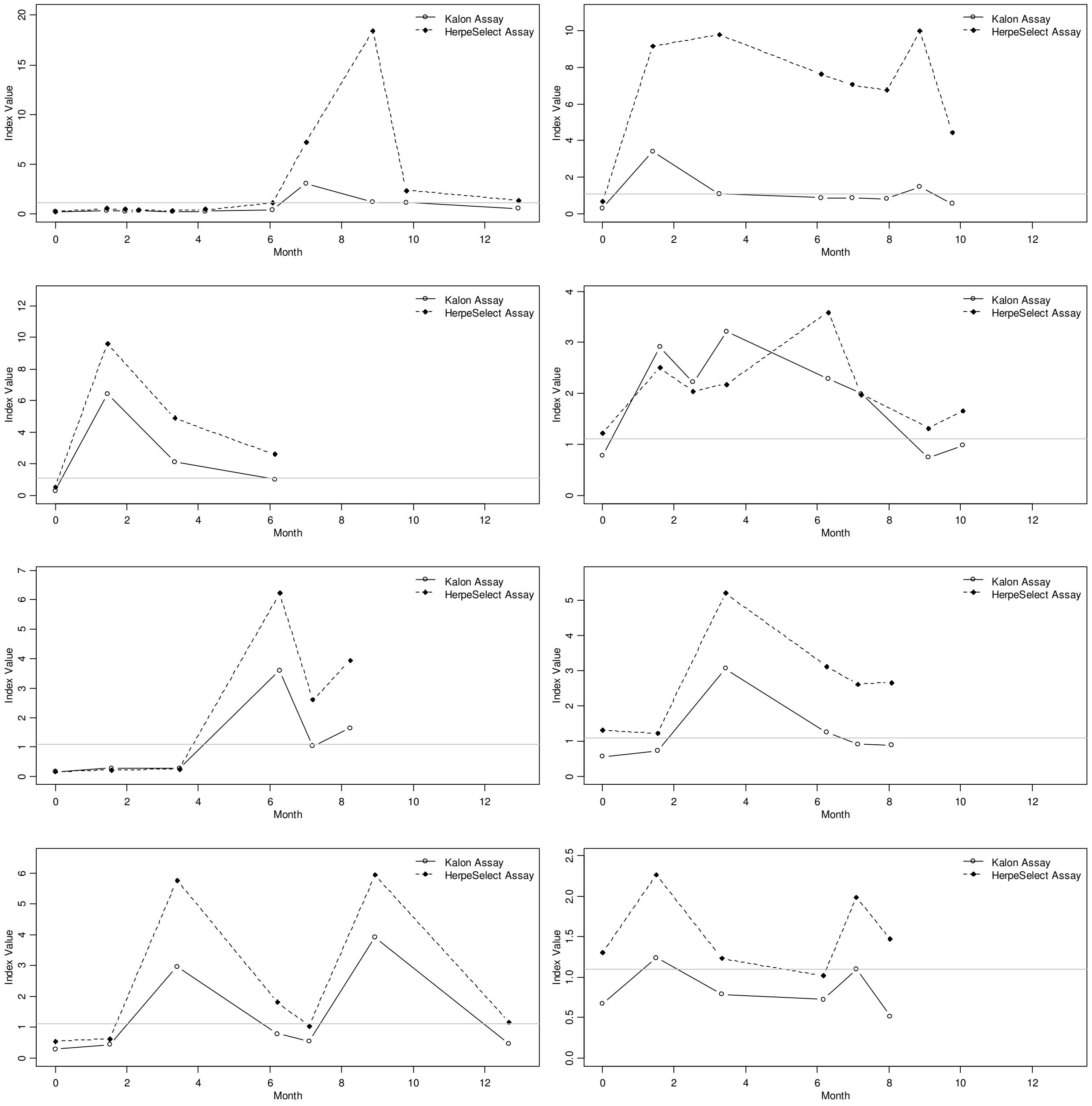
Kalon Assay
HerpeSelect Assay
Index Value
Month

Nr°	BASELINE (index values)		FINAL VISIT		POSITIVITY (sero-positive in both assays)		LOWEST VALUE AFTER POSITIVITY	
	Kalon	HerpeSelect	Kalon	HerpeSelect	Kalon	HerpeSelect	Kalon	HerpeSelect
1	0.173	0,249	1,242	2,116	3,364	3,718	**0,198**	*1,031*
2	0,556	1,174	0,961	2,942	3,101	6,315	*0,961*	2,942
3	0,221	0,480	0,913	2,095	1,873	3,942	**0,697**	1,308
4	0,342	2,315	0,756	1,815	7,426	15,306	**0,756**	1,655
5	0,994	0.944	2.949	1,961	1,886	1,669	**0,896**	*0,984*
6	0,213	0,194	0,772	1,142	1,560	3,193	**0,772**	1,142
7	0,218	0,283	1,128	2,051	2,427	3,230	**0,485**	*0,977*
8	0,157	0,428	0,794	2,213	1,578	3,502	**0,794**	2,213
9	0,225	0,280	0,773	1,718	3,063	7,246	**0,552**	1,387
10	0,294	0,703	0,560	4,473	3,427	9,163	**0,560**	4,473
11	0,298	0,517	1.005	2.602	6,391	9,607	*1,005*	2,602
12	0,776	1,212	0,983	1,650	2,908	2,498	**0,741**	1,313
13	0,163	0,174	1,636	3,943	3,598	6,221	*1,025*	2,600
14	0,562	1,315	0,896	2,673	3,065	5,208	**0,896**	2,673
15	0,275	0,543	0,456	1,168	2,954	5,748	**0,456**	*1,040*
16	0,672	1,304	0,511	1,475	1,238	2,266	**0,511**	*1,015*

Index values of the 16 cases that had a sudden decrease in index values. IV are provided at baseline, final visit, visit of dual positivity in both assays and the lowest IV after positivity.
Bold: IV<0.90 (sero-negative according to manufacturer's guidelines)
Italics: 0.90≥iv≤1.10 (equivocal result according to manufacturer's guidelines)
Highlighted: IV<0.66 (not sero-positive according to the lowered cut-off)
