# Supplementary material for: Prevalence and Incidence Estimation of HSV-2 by Two IgG ELISA Methods among South African Women at High Risk of HIV
Source: PLoS One. 2015 Mar 23;10(3):e0120207. doi: 10.1371/journal.pone.0120207 (PMC4370866; doi:10.1371/journal.pone.0120207)
Supplement: S1 Table — (PDF) [file pone.0120207.s003.pdf]

S2: Time of seroconversion for HerpeSelect and Kalon assay using manufacturer's cut-off and lowered cut-off

ID	Time of Seroconversion (days)			Difference in Seroconversion Time (weeks)		
	Kalon (IV>1.10)	Kalon Lowered Cut-off (IV>0.66)	HerpeSelect (IV>1.10)	Δ Kalon - HerpeSelect	Δ Kalon Lowered - HerpeSelect	Δ Kalon - Kalon Lowered
1	381+	381+	75	44+	4+	0
2	294+	294+	22	39+	39+	0
3	378+	378+	190	27+	27+	0
4	379+	379+	226	22+	22+	0
5	99+	99+	50	7+	7+	0
6	272+	272+	230	6+	6+	0
7	242+	242+	227	2+	2+	0
8	389+	197	155	33+	6	27+
9	243+	24	24	31+	0	31+
10	213+	25	25	27+	0	27+
11	325+	199	143	26+	8	18+
12	185+	23	23	23+	0	23+
13	379+	379	239	20+	20	0+
14	217+	109	109	15+	0	15+
15	379+	324	324	8+	0	8+
16	155+	127	127	4+	0	4+
17	254	22	22	33	0	33
18	311	238	141	24	14	10
19	142	142	22	17	17	0
20	142	142	22	17	17	0
21	142	142	62	12	12	0
22	143	71	71	10	0	10
23	75	24	24	7	0	7
24	157	157	114	6	6	0
25	113	113	71	6	6	0
26	286	202	244	6	-6	12
27	22	22	22	0	0	0
28	23	23	23	0	0	0
29	24	24	24	0	0	0
30	26	26	26	0	0	0
31	71	71	71	0	0	0
32	71	71	71	0	0	0
33	72	72	72	0	0	0
34	73	73	73	0	0	0
35	74	74	74	0	0	0
36	111	111	111	0	0	0
37	141	141	141	0	0	0
38	142	142	142	0	0	0
39	142	142	142	0	0	0
40	143	143	143	0	0	0

ID	Time of Seroconversion (days)			Difference in Seroconversion Time (weeks)		
	Kalon (IV>1.10)	Kalon Lowered Cut-off (IV>0.66)	HerpeSelect (IV>1.10)	Δ Kalon - HerpeSelect	Δ Kalon Lowered - HerpeSelect	Δ Kalon - Kalon Lowered
41	144	144	144	0	0	0
42	146	146	146	0	0	0
43	147	147	147	0	0	0
44	148	148	148	0	0	0
45	169	169	169	0	0	0
46	204	204	204	0	0	0
47	240	240	240	0	0	0
48	255	255	255	0	0	0
49	287	287	287	0	0	0
50	295	295	295	0	0	0
51	320	320	320	0	0	0
52	244+	25				31
53	70+	36				5
54	295+	22				39
55	22					
56	24					
57	25					
58	34					
59	73					
60	75					
61	130					
